# Long-duration effect of multi-factor stresses on the cellular biochemistry, oil-yielding performance and morphology of *Nannochloropsis oculata*

**DOI:** 10.1371/journal.pone.0174646

**Published:** 2017-03-27

**Authors:** Likun Wei, Xuxiong Huang

**Affiliations:** 1 Key Laboratory of Genetic Resources for Freshwater Aquaculture and Fisheries, College of Fisheries and Life Science, Shanghai Ocean University, Shanghai, China; 2 Shanghai Engineering Research Centre of Aquaculture, Shanghai, China; 3 Shanghai University Knowledge Service Platform, Shanghai Ocean University Aquatic Animal Breeding Centre (ZF1206), Shanghai, China; Leibniz-Institut fur Pflanzengenetik und Kulturpflanzenforschung Gatersleben, GERMANY

## Abstract

Microalga *Nannochloropsis oculata* is a promising alternative feedstock for biodiesel. Elevating its oil-yielding capacity is conducive to cost-saving biodiesel production. However, the regulatory processes of multi-factor collaborative stresses (MFCS) on the oil-yielding performance of *N*. *oculata* are unclear. The duration effects of MFCS (high irradiation, nitrogen deficiency and elevated iron supplementation) on *N*. *oculata* were investigated in an 18-d batch culture. Despite the reduction in cell division, the biomass concentration increased, resulting from the large accumulation of the carbon/energy-reservoir. However, different storage forms were found in different cellular storage compounds, and both the protein content and pigment composition swiftly and drastically changed. The analysis of four biodiesel properties using pertinent empirical equations indicated their progressive effective improvement in lipid classes and fatty acid composition. The variation curve of neutral lipid productivity was monitored with fluorescent Nile red and was closely correlated to the results from conventional methods. In addition, a series of changes in the organelles (e.g., chloroplast, lipid body and vacuole) and cell shape, dependent on the stress duration, were observed by TEM and LSCM. These changes presumably played an important role in the acclimation of *N*. *oculata* to MFCS and accordingly improved its oil-yielding performance.

## Introduction

Microalgae are a diverse group of microorganisms with various unique biological characteristics, including high photosynthetic energy transfer efficiency, high biomass productivity, excellent adaptability to various environments and capability of producing a broad variety of bioenergy [1–3]. For example, the members of *Nannochloropsis* genus are marine Eustigmatophyceae microalgae with advantages such as fast growth and easy cultivation, capable of storing large triacylglycerols (TAGs) under particular culture conditions and are therefore an environmentally friendly biodiesel feedstock with great developmental potential [4]. However, microalgae biodiesel still faces many problems in commercial production, such as high oil-yielding species to be screened or constructed, and technological innovations in large-scale cultivation, harvesting, oil extraction and transesterification of biolipids to biodiesel [1].

Increasing the oil-yielding capacity of microalgae is one of the keys to reduce the production cost of microalgae biodiesel, by improving both metabolic efficiency in lipid biosynthesis pathways and biomass productivity of microalgae [5]. There has been progress in increasing the lipid biosynthesis capability of microalgae through high biomass cultivation, metabolic engineering and genetic engineering [6–8]. To increase fatty acid (FA) biosynthesis, genetic engineering regulating cell metabolic pathways is feasible, including the over-expression or inhibition of certain rate-limiting enzymes [7–9], the comprehensive regulation of lipid biosynthesis by transcription factors [10], the construction of genes in association with spontaneous secretion of FA and the construction of efficient expression vectors for exogenous genes [3].

Metabolic pathway regulation, however, is relatively easy to undertake, the nature of which is to expose microalgae to environmental stresses, thus resulting in cellular metabolic flux with a shift to lipid biosynthesis [3, 6]. Microalgae have evolved countermeasures for survival in the face of various environmental adversities [6, 11]. The adaptation generated can quickly become evident as variations in their growth, physiology, biochemistry and morphology. However, high diversity of microalgae may well lead to the differences in cellular metabolic pathways and metabolic regulation between different species [6, 12, 13]. It is therefore possible to regulate many physiological metabolic processes in microalgae by changing their culture conditions, to obtain the end products or intermediate products of algal cells, such as proteins, FAs, pigments and polysaccharides.

By the orthogonal experiments of both multi-factor and multi-level stresses, we obtained the optimal culture conditions for *N*. *oculata* with a high TL content [14]. However, little information is available on the regulatory processes of multi-factor collaborative stresses (MFCS) on the oil-yielding performances of *N*. *oculata*. This study continued to adapt the optimal culture method for metabolic regulation of *N*. *oculata*. We explored the variations in growth, cellular biochemistry, FA and TAG production, biodiesel property and morphology of *N*. *oculata* during MFCS (i.e., high irradiation, nitrogen deficiency, and elevated iron supplementation). A rapid and high-throughput method using Nile red (NR) staining for monitoring neutral lipid (NL) in *N*. *oculata* was established; we dynamically detected the relative content of NL, and quickly and effectively determined the optimal harvest period to obtain the highest lipid productivity. Through LSCM and TEM, we synthetically observed the variations in the lipid bodies and cellular ultrastructure of *N*. *oculata* during the stresses, which confirmed the lipid accumulation process.

## Materials and methods

### Culture of microalgae

*Nannochloropsis oculata* was provided by the Culture Collection of Microalgae at Shanghai Ocean University. This microalgal strain was first axenically cultivated in 60-L photobioreactors under favourable conditions: f/2 culture medium, correct nutrient concentrations, 20 of salinity, 25°C, 150 μmol·m^-2^·s^-1^ of continuous irradiation, and 0.2 vvm of continuous sterile aeration.

When NO_3_-N concentration in the microalgal suspension as described above was lower than 2 mg N·mL^-1^ (12.32 mg N·mL^-1^ in f/2 medium), it was supposed to be in nitrogen deficiency conditions. *N*. *oculata* was then immediately and directly exposed to optimal culture conditions for lipid storage: 360 μmol·m^-2^·s^-1^ of continuous elevated irradiation and 6.72 mg Fe·L^-1^ of elevated ferric citrate supplementation. This combined light-nitrogen-iron stress had been demonstrated as effectively regulating lipid metabolism in *N*. *oculata* [14]. In addition, this process does not use the conventional centrifugation-resuspension method and thus saves energy. Microalgae were cultivated in their original 60-L photobioreactors (60 L of actual liquid volume) for an 18-d duration and were kept in circular flow with the help of a mini-submersible pump and water-circulating pipe. Other culture conditions at that moment were 6.185×10^7^ cells·mL^-1^ of microalgal cell density, 20‰ of salinity, 20°C, 1.45±0.07 mg N·mL^-1^ of NO_3_-N concentration, 0.24±0.00 mg P·mL^-1^ of total phosphorus concentration, and 0.2 vvm of continuous sterile aeration.

### Growth performances

Microalgal cell density and biomass concentration were determined everyday by haemocytometer and gravimetric methods, respectively. First, 20–40 mL (V) of microalgal suspension was filtered through an 0.44 μm pre-weighed cellulose acetate membrane (W_0_), then cleaned by an equivalent volume of deionized water (pH = 4), and finally dried at 105°C until a constant weight (W_1_). After that, the biomass concentration was determined as (g·L^-1^ DW) = (W_1_−W_0_)/V.

### Assays of pigment, protein and sugar

The contents of pigment, protein and sugar were determined every other day in accordance with Pruvost’s method [15]. Pigment (e.g., chl *a* and carotenoids) contents were obtained by spectrophotometric methods and total sugar content was determined by phenol-sulphuric acid method. To assay protein content, 4 mL of microalgal culture was harvested and re-suspended in 1 mL NaOH at 2 mol·L^-1^. The samples were placed at 95°C for 10 mins and then neutralized by adding 1 mL HCL of 1.6 mol·L^-1^. The supernatant obtained by centrifugation was used to analyse protein content by the Folin-phenol method.

### Lipid analysis

Enough microalgal suspension was harvested by centrifugation and washed 3 times with deionized water (pH = 4). The cell pellets were then freeze-dried into powder at −46°C in preparation for the determination of lipid analysis every other day.

Total lipid (TL) content was determined by the chloroform-methanol (2:1, v/v) method [16]. The fatty acid (FA) profile was analysed by Aglient 7890A-5975C GC-MS using the direct transesterification method [17]. Lipid class compositions were assayed by a thin layer chromatography–flame ionization detector (TLC-FID) method. First, the dried TL was dissolved in moderate chloroform at 10–20 mg·mL^-1^. The solution was then spotted into a silicon rod (10 mm×0.32 mm) for chromatography, which was then subjected to an outspread procedure with a solvent system of n-hexane-ether-formic acid (42:28:0.3, v:v:v). After drying, the silicon rod was used for analysis by IATROSCAN MK-6s (IATRON LABORATORIES INC., Japan). The resulting chromatogram was processed with Chormstar (IATRON) according to the qualitative analysis of the standard substances from Sigma for phospholipids (PLs), triacylglycerols (TAGs), diacylglycerol, monoacylglycerol, free FA, and cholesterol. Finally, the area normalization method was adopted to calculate the relative proportions of lipid classes.

### Evaluation of biodiesel properties

According to previous studies, there exist mathematically relational models between the molecular structure and profile of fatty acid methyl ester (FAME) and biodiesel properties [18]. Therefore, we can predict the properties of microalgal biodiesel (i.e., viscosity, iodine value, cetane number, and cold filter plugging point) by the relevant empirical equations below, helpful for evaluating whether it is an alternative to fossil fuel.

The 3 specific parameter definitions are:

Relative chain length (RCL),
  $$\text{RC}L=\sum N_{i}\times P_{i};$$ (1)
Saponification value (SV, mg KOH·g^-1^ oil),
  $$SV=\sum \left(560\times P_{i}\right)/MW_{\text{i}};$$ (2)
Long chain saturated factor (LCSF),
  $$LCSF=0.1\times P_{C16:0}+0.5\times P_{C18:0}+P_{C20:0}+1.5\times P_{C22:0}+2\times P_{C24:0}.$$ (3)

The formulae of the 4 prediction models are:

Viscosity (Vis, mm^2^·s^-1^) [19],
  $$Vis=-1.72955+0.31247\times \text{RC}L+0.04228\times P_{C22:1};$$ (4)
Iodine value (IV, g I_2_·100g^-1^ oil) [20],
  $$IV=\sum \left(254\times m\times P_{i}\right)/MW_{i};$$ (5)
Cetane number (CN) [21],
  CN=46.3+5458/SV−0.225×IV; (6)
Cold filter plugging point (CFPP, °C) [22],
  CFPP=3.1417×LCSF−16.477. (7)

For these formulae, *i*, *N*, *m*, *P*, and *MW* indicate certain FAME, the carbon chain length of FAME, the double bond number of FAME, the relative percentage content of FAME (% total FAMEs), and the molecular weight of FAME, respectively.

### Nile red staining and quantification of neutral lipids

Nile red (NR) staining conditions optimized for *N*. *oculata* were as follows. The final solution was 3 mL, containing a certain volume of microalgal suspension, 0.5 ug/mL of NR, and 15% (v:v) of DMSO. The microalgal cell density in the final solution was diluted to 1.0×10^7^ cells·mL^-1^, or keeping its original state (6.0–8.5×10^7^ cells·mL^-1^), both situations of them taken into account. The solution was then incubated at 40°C for 10 mins before determinations. If the lipid content was very high when in the original state, the staining time was extended to 15 mins.

After the pre-treatment as described above, the fluorescence intensity (FI) of cellular neutral lipids (NLs) in the solution stained with NR was determined every day using a Fluorescence Spectrophotometer (HITACHI F-4600 FL). Its parameter settings were 523 nm of excitation wavelength, and 578 nm of emission wavelength. Eventually, the relative content of NL was expressed as FI (arbitrary units, a.u.) for each day, in line with its value under 1.0×10^7^ cells·mL^-1^ of stained microalgal suspension. The relative productivity of NL was equal to the quotient of FI under 6.0–8.5×10^7^ cells·mL^-1^ divided by the stress duration. The maximal volumetric NL productivity was considered as an indicator of the optimal harvest period for the microalgae.

### Lipid bodies microexamination and cellular ultrastructure

Intracellular lipid bodies (LBs) stained with NR were microscopically observed by LSCM (ZEISS LSM 710) every other day. In addition, to observe the cellular ultrastructures at different stress durations (day 0, 4, 8, 12, 14, 16, and 18), an appropriate quantity of microalgal cell pellet was involved in a series of bio-specimen preparation procedures before observed by TEM (PHILIP CM-120), including fixation, dehydration, clearing, impregnation, embedding, trimming, cutting and staining.

### Data analysis and processing

All sample indices underwent parallel determinations 3 times. The results were expressed as the means±SD, and analysed using one-way ANOVA and Duncan’s new multiple-range test for statistical significance by PASW Statistics18.0 at a significance level *P*<0.05. Images from LCSM and TEM were processed by their accessory image processing systems. In addition, the correlation and regression analyses between FI and the results obtained from lipid analyses were also conducted, as well as the variance analysis of the regression model.

## Results

### Cell growth performance

**Fig 1:**
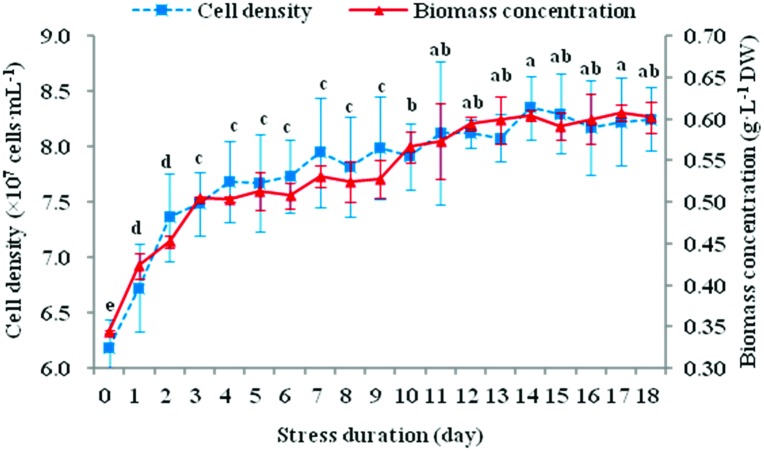
Variation in the cell growth of *N*. *oculata* during long-term MFCS. *N*. *oculata* was cultured axenically in 60-L photobioreactors with continuous sterile aeration, under multi-factor collaborative stress (MFCS; i.e., 360 μmol·m^-2^·s^-1^ of high irradiation, nitrogen deficiency and 6.72 mg Fe·L^-1^ of iron supplementation). The cell density and biomass concentration represent the cell growth performances of *N*. *oculata* in quantity and weight, respectively. Different small letters for biomass concentration denote significant differences among different stress durations at *P*<0.05. (means±SD of three replicates).

Microalgal cells began to grow slowly after day 3 (Fig 1). Biomass concentration was not significantly different from days 3–9 or days 11–18 (*P*>0.05). Cell density on day 14 (8.351×10^7^ cells·mL^-1^, the maximum value) increased by 35.0% compared to the initial value (6.185×10^7^ cells·mL^-1^), while biomass concentration increased by 75.6% correspondingly (from 0.344 g·L^-1^ to 0.604 g·L^-1^), presumably due to the increased cellular volume and cellular specific gravity observed from microscopy. The storage carbon/energy reservoir in a single cell therefore seemed to increase. It was thus evident that *N*. *oculata* retained the ability to grow slowly during a short period of environmental stress.

### Cellular biochemistry variation

**Fig 2:**
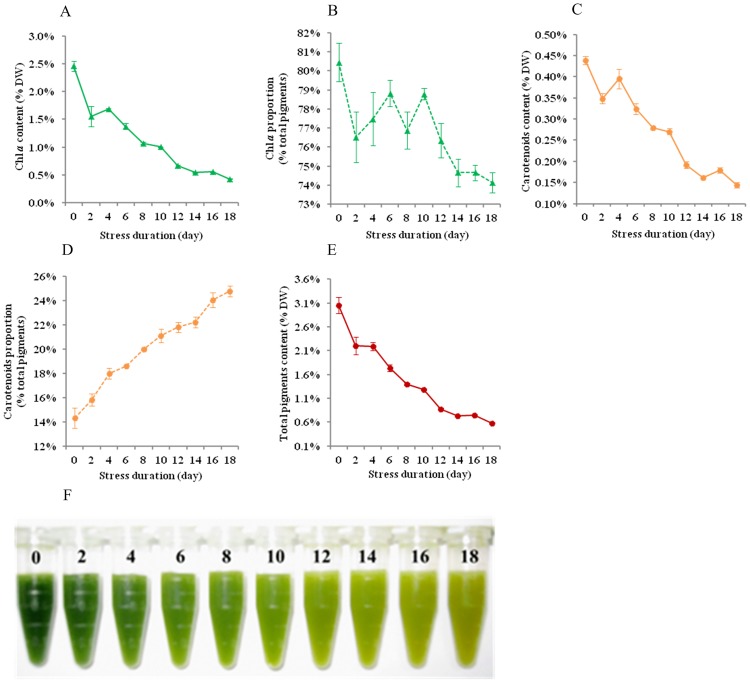
Variations in the pigment contents and cell suspension colour of *N*. *oculata* during long-term MFCS. MFCS means multi-factor collaborative stresses (i.e., 360 μmol·m^-2^·s^-1^ of high irradiation, nitrogen deficiency and 6.72 mg Fe·L^-1^ of iron supplementation). The solid lines denote the absolute contents (% DW) of chl *a* (A), carotenoids (C) and total pigments (E). The dotted lines denote the relative contents (% total pigments) of chl *a* (B) and carotenoids (D). (F) Colour changes of *N*. *oculata* suspension, concentrated 5 times by centrifugation (5 mL re-suspended in 1 ml); the numbers denote the stress duration (day). (means±SD of three replicates).

The photosynthetic pigments of *N*. *oculata* were mainly chl *a* and carotenoids, with the initial values accounting for 84.86% and 15.14% of total pigments, respectively (Fig 2B and 2D). The carotenoids proportion (% total pigments) on day 18 increased by 72.5% compared to its initial value, but it was the opposite for chl *a*. As a result, the colour of the algal cell suspension gradually changed from green to yellow (Fig 2F). Chl *a* content (% DW) declined substantially under MFCS, as did carotenoids (Fig 2A and 2C). Finally, the total pigment content (% DW) on day 18 decreased by 80.96% compared to the initial value (Fig 2E).

**Fig 3:**
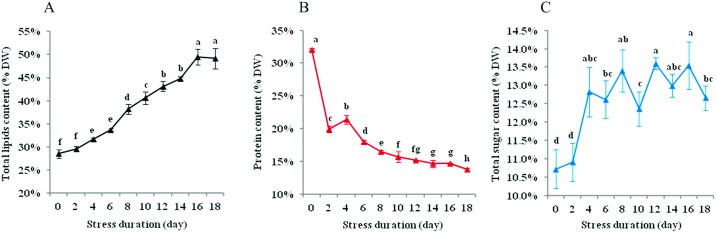
Content variations in the total lipids (A), protein (B) and total sugar (C) of *N*. *oculata* during long-term MFCS. MFCS means multi-factor collaborative stresses (i.e., 360 μmol·m^-2^·s^-1^ of high irradiation, nitrogen deficiency and 6.72 mg Fe·L^-1^ of iron supplementation). Different small letters in one panel denote significant differences among different stress durations at *P*<0.05. (means±SD of three replicates).

TL (% DW) showed an increasing trend, from 28.52% to 49.14% on day 18 (Fig 3A). The protein content (% DW) declined remarkably during the first 2 d of MFCS and then decreased continuously, from 32.10% to 13.76% on day 18 (Fig 3B). Interestingly, the total sugar content (% DW) increased from 10.73% to 12.82% on day 4, but then fluctuated between 12.37% and 13.59% (Fig 3C).

### Lipid class composition, FA profile, and biodiesel properties

**Fig 4:**
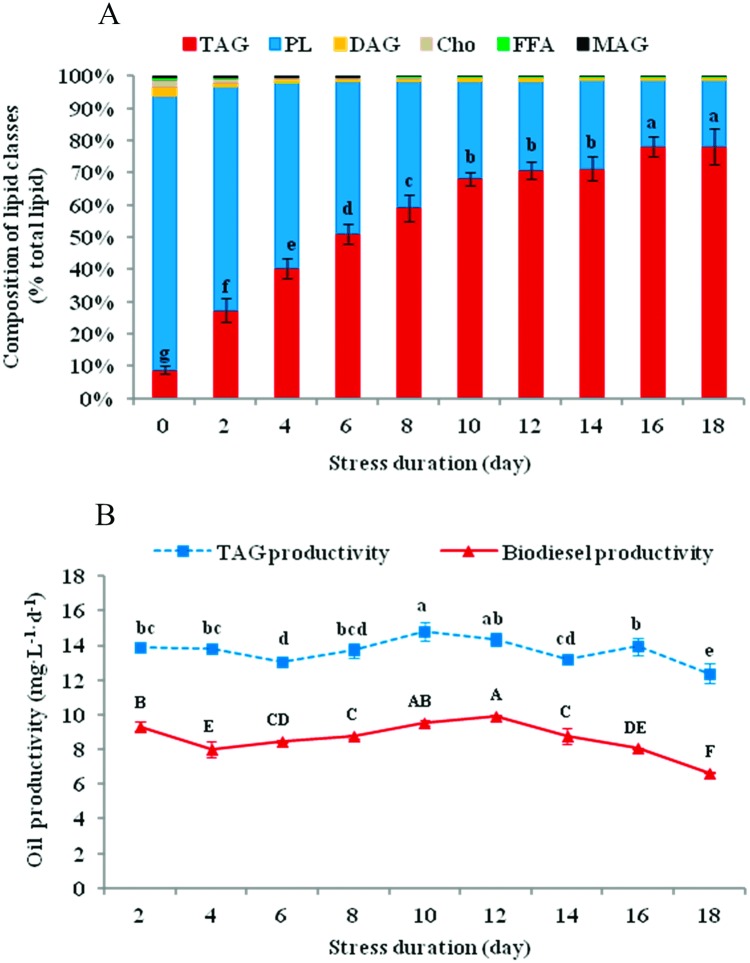
Variations in the lipid classes composition (a) and oil productivity (b) of *N*. *oculata* during long-term MFCS. MFCS means multi-factor collaborative stresses (i.e., 360 μmol·m^-2^·s^-1^ of high irradiation, nitrogen deficiency and 6.72 mg Fe·L^-1^ of iron supplementation). TAG, triacylglycerol; PL, phospholipid; DAG, diacylglycerol; Cho, cholesterol; FFA, free fatty acids; MAG, monoacylglycerol. Biodiesel productivity indicates the productivity of total fatty acid methyl esters. See S1 Fig for the neutral and polar lipid compositions included in this analysis. Different small or capital letters in one panel denote significant differences within the same type of letter among different stress durations at *P*<0.05. (means±SD of three replicates).

Similar to the TL content, the TAG proportion (% TL) increased from 8.85% to 78.16% on day 18, while the phospholipid (PL) proportion decreased from 84.97% to 20.60%, and the other minor lipid classes (i.e., diacylglycerol, monoacylglycerol, free FA and cholesterol) proportions all underwent a significant decline (Fig 4A).

The FA profiles of *N*. *oculata* mainly contained 16:0, 16:1n7, 18:1n9, 20:5n3 (eicosapentaenoic acid, EPA), and 20:4n6 (arachidonic acid, ARA) (Table 1), similar to the majority species of the Eustigmatophyceae [23]. Along with the stresses, the proportions of saturated FA (SFA) and monounsaturated FA (MUFA) (% total FA) gradually increased, from a sum of 63.56% to 92.18%, while the polyunsaturated FA (PUFA, including EPA and ARA) proportion increasingly declined, from 21.29% to 4.12%. Conceivably, if the culture had continued after day 18, the reduction in PUFA proportions might have continued. As in the previous nitrogen-iron-temperature combined culture experiment, the EPA proportion could finally drop as low as 1% [14]. In particular, both the proportion (% total FA, Table 1) and absolute content (mg·g^-1^ DW, S1 Table) of 16:0 increased, as well as that of 18:1n9, which suggests that they play a conspicuous role in the course of TAG reserve. In addition, the total FA content (i.e., biodiesel yield) increased from 110.21 to 261.94 mg·g^-1^ (Table 1).

**Table 1:** Variation in the fatty acid profile of *N*. *oculata* during long-term MFCS.

Fatty acids (% total FA)	Stress duration (day)									
	0	2	4	6	8	10	12	14	16	18
14:0	3.22±0.26^a^	2.67±0.12^bc^	2.84±0.62^b^	3.12±0.24^a^	2.82±0.08^b^	2.58±0.08^bc^	2.53±0.07^c^	2.62±0.11^bc^	2.58±0.08^bc^	2.55±0.24^c^
16:0	24.06±0.54^g^	32.41±1.28^f^	36.43±1.73^e^	40.01±0.46^d^	41.11±0.39^abc^	40.68±0.21^bcd^	41.57±0.68^a^	41.39±0.61^ab^	40.48±0.24^cd^	41.11±0.24^abc^
16:1n7	26.64±1.23^d^	28.91±2.14^abc^	28.54±2.63^abc^	26.80±0.35^d^	28.04±0.24^c^	29.27±0.34^ab^	29.05±0.69^abc^	28.27±0.43^bc^	28.29±0.34^bc^	29.66±1.06^a^
18:0	2.08±0.02^a^	1.37±0.04^cd^	1.38±0.07^cd^	1.43±0.12^cd^	1.38±0.10^cd^	1.36±0.06^cd^	1.46±0.09^bc^	1.46±0.13^bc^	1.56±0.06^b^	1.31±0.12^d^
18:1n9	5.78±0.08^g^	8.38±0.79^f^	8.44±0.23^f^	10.57±0.33^e^	12.28±0.32^d^	14.10±0.39^c^	14.43±0.51^c^	15.49±0.22^b^	16.60±0.12^a^	16.47±0.11^a^
18:2n6	5.46±0.01^a^	3.86±0.35^b^	4.15±1.16^b^	2.71±0.06^c^	2.26±0.03^d^	1.98±0.06^d^	1.64±0.12^e^	1.59±0.18^e^	1.61±0.04^e^	1.46±0.12^e^
ARA	9.69±0.60^a^	7.20±0.04^b^	5.81±0.68^c^	4.98±0.23^d^	3.91±0.18^e^	3.06±0.10^f^	2.94±0.17^fg^	2.93±0.09^fg^	2.74±0.06^g^	2.25±0.24^h^
EPA	21.29±0.10^a^	13.36±0.14^b^	10.70±0.24^c^	8.73±0.26^d^	6.73±0.29^e^	5.63±0.24^f^	5.19±0.24^g^	5.04±0.26^g^	4.95±0.21^g^	4.12±0.65^h^
Others	1.79±0.17^ab^	1.84±0.08^a^	1.72±0.23^bc^	1.64±0.08^c^	1.47±0.04^d^	1.35±0.04^e^	1.20±0.02^f^	1.20±0.04^f^	1.19±0.10^f^	1.08±0.03^g^
∑16C	50.70±0.69^i^	61.32±0.86^h^	64.97±0.91^g^	66.81±0.73^f^	69.15±0.35^de^	69.95±0.53^bc^	70.62±0.01^ab^	69.67±0.60^cd^	68.77±0.10^e^	70.77±0.81^a^
∑18C	13.32±0.09^g^	13.61±0.48^hg^	13.96±0.86^h^	14.71±0.50^f^	15.91±0.32^e^	17.43±0.48^d^	17.53±0.48^d^	18.54±0.43^c^	19.76±0.22^a^	19.24±0.13^b^
∑20C	30.98±0.51^a^	20.56±0.18^b^	16.50±0.92^c^	13.72±0.49^d^	10.64±0.36^e^	8.69±0.21^f^	8.13±0.40^g^	7.97±0.34^g^	7.70±0.14^g^	6.36±0.89^h^
∑SFA	30.42±0.67^d^	37.69±1.54^c^	41.91±2.68^b^	45.85±0.42^a^	46.47±0.40^a^	45.69±0.26^a^	46.50±0.66^a^	46.44±0.44^a^	45.58±0.28^a^	45.83±0.16^a^
∑MUFA	33.14±1.18^f^	37.90±1.37^e^	37.44±2.44^e^	37.72±0.47^e^	40.63±0.53^d^	43.64±0.18^c^	43.73±1.19^c^	44.01±0.55^c^	45.11±0.18^b^	46.35±1.17^a^
∑PUFA	36.44±0.50^a^	24.42±0.17^b^	20.65±0.24^c^	16.43±0.49^d^	12.90±0.33^e^	10.67±0.20^f^	9.77±0.53^g^	9.56±0.48^g^	9.30±0.10^g^	7.82±1.01^h^
∑FAMEs*	110.21±0.50^h^	125.20±5.07^g^	138.95±16.32^f^	174.83±2.78^e^	205.81±5.98^d^	235.54±7.45^c^	264.51±0.53^ab^	266.63±25.86^ab^	277.90±1.93^a^	261.94±0.64^b^

Note: MFCS means multi-factor collaborative stresses (i.e., 360 μmol·m^-2^·s^-1^ of high irradiation, nitrogen deficiency and 6.72 mg Fe·L^-1^ of iron supplementation). Other FAs (i.e., others in this table) are C12:0, C15:0, C17:0 and C17:1n7, all of whose contents are less than 1% of total FAs. ARA, arachidonic acid (C20:4n6); EPA, eicosapentaenoic acid (C20:5n3); SFA, saturated FA; MUFA, monounsaturated FA; PUFA, polyunsaturated FA.

* The unit of ∑FAMEs is mg·g^-1^ DW. See S1 Table for the absolute quantification of FA profile (mg·g^-1^ DW). Different superscript letters in the same row denote significant differences at *P*<0.05. (means±SD of three replicates).

The productivities of TAG and biodiesel reached the maximums of 14.83 and 9.94 mg·L^-1^·d^-1^ on day 10 and 12, respectively (Fig 4B), which could be a reference for the best time to harvest. Based on the pertinent empirical equations as described in the experimental procedures, the variation in the biodiesel properties of *N*. *oculata* during MFCS could be predicted (Table 2). Both Vis (3.46–3.72 mm^2^·s^-1^) and CFPP (−5.65 to −1.31°C) could meet the biodiesel standards of the USA and the European Union throughout the culture period, while IV (72.67–156.80 g·100g^-1^) and CN (38.76–56.88) could meet the standards after day 4 and increasingly improved as the MFCS continued (Table 2 and S2 Table).

**Table 2:** Variation in the biodiesel properties of *N*. *oculata* during long-term MFCS.

Biodiesel properties	Stress duration (day)									
	0	2	4	6	8	10	12	14	16	18
Vis (mm^2^·s^-1^)	3.72±0.00^a^	3.60±0.00^b^	3.55±0.00^c^	3.51±0.01^d^	3.48±0.00^e^	3.47±0.01^e^	3.46±0.00^ef^	3.47±0.01^e^	3.47±0.00^e^	3.45±0.01^f^
IV (gI_2_·100g^-1^)	156.80±0.41^a^	118.49±1.28^b^	103.41±1.21^c^	90.49±1.52^d^	80.84±1.03^e^	75.91±0.77^f^	73.23±0.61^g^	72.67±1.20^g^	72.71±0.74^g^	68.68±2.49^h^
CN	38.76±0.08^h^	46.97±0.31^g^	50.19±0.27^f^	52.99±0.32^e^	55.07±0.22^d^	56.14±0.16^c^	56.73±0.13^b^	56.87±0.25^b^	56.88±0.17^b^	57.72±0.51^a^
CFPP (°C)	-5.65±0.20^f^	-4.14±0.47^e^	-2.87±0.65^d^	-1.66±0.15^c^	-1.39±0.17^abc^	-1.57±0.05^bc^	-1.13±0.07^a^	-1.18±0.10^a^	-1.31±0.16^ab^	-1.51±0.26^bc^

Note: MFCS means multi-factor collaborative stresses (i.e., 360 μmol·m^-2^·s^-1^ of high irradiation, nitrogen deficiency and 6.72 mg Fe·L^-1^ of iron supplementation). The estimation of four biodiesel properties was based on the pertinent empirical equations from the fatty acid methyl esters profile. Vis, viscosity; IV, iodine value; CN, cetane number; CFPP, cold filter plugging point. Different superscript letters in the same row of each treatment denote significant differences at *P*<0.05. (means±SD of three replicates).

### Relative content and relative productivity of NL

**Fig 5:**
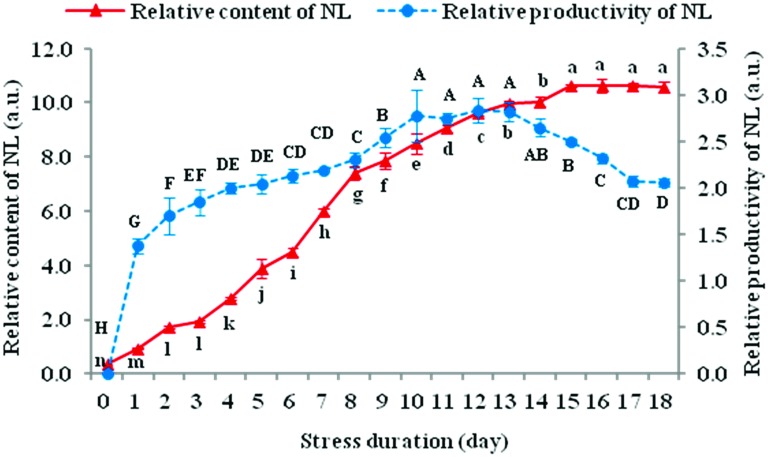
Variations in the relative content and relative productivity of Neutral Lipids (NLs) in *N*. *oculata* during long-term MFCS. MFCS means multi-factor collaborative stresses (i.e., 360 μmol·m^-2^·s^-1^ of high irradiation, nitrogen deficiency and 6.72 mg Fe·L^-1^ of iron supplementation). The relative content of NL was represented by the fluorescence intensity (FI, a.u.) of cellular NL stained with Nile red in each day, as the stained *N*. *oculata* suspension was diluted to a cell density of 1.0×10^7^ cells·mL^-1^. The relative productivity of NL was represented by the quotient of FI in each day divided by stress duration, as the stained *N*. *oculata* suspension was at its original cell density of 6.0–8.5×10^7^ cells·mL^-1^. See S2 Fig for details on the relative production of NL included in this analysis. Different small letters for relative content of NL and capital letters for relative productivity of NL mean significant differences within the same type of letter among different stress durations at *P*<0.05. (means±SD of three replicates).

The fluorescence intensity (FI) of microalgal cells stained with NR was distinct in different stress durations (Fig 5). The relative content of neutral lipid (NL), that is FI, was drastically augmented as the stress progressed, reaching its peak on day 15, and had no significant differences during days 15–20. It was basically consistent with the results obtained from traditional gravimetric methods [16] (see Lipid analysis in Materials and methods), such as TL content (maximum 49.47% DW, day 16) and TAG content (maximum 38.61% DW, day 16). The relative productivity of NL showed a tendency to increase at first and then to decrease, and reached its maximum during days 10–13 (Fig 5). It was compatible with TAG productivity (maximum 14.83 mg·L^-1^·d^-1^, day 10) and biodiesel productivity (maximum 9.94 mg·L^-1^·d^-1^, day 12) (Fig 4B).

The FI of microalgal cells stained with NR had significant linear positive correlations with TL content or yield obtained by the chloroform-methanol method [16], the TAG content or yield obtained by the TLC-FID method, and the biodiesel yield obtained by the GC-MS method [17], with correlation coefficients between 0.971–0.997, *P*<<0.01 (Table 3). Similarly, the regression equations had high fitting degrees, with determination coefficients of 0.944–0.993, *P*<<0.01. Therefore, by determining the FI of microalgal cells stained with NR, it might comparatively accurately estimate the oil-yielding capacity of *N*. *oculata* by means of relevant linear regression equations.

**Table 3:** Regression results of the oil-yielding capacity and fluorescence intensity of cellular neutral lipids in *N*. *oculata* using Nile red staining.

Regression variables	Regression equations	*r*	*R*^*2*^	Sig.
TL content ^a^ (% DW)	y = 0.0193x + 0.2612	0.971	0.944 (0.936)	0.00000284
TAG content ^a^ (% DW)	y = 0.0316x + 0.0216	0.986	0.972 (0.969)	0.00000016
Biodiesel yield ^a^ (mg·g^-1^ DW)	y = 16.325x + 98.289	0.994	0.987 (0.986)	0.00000001
TL yield ^b^ (mg·L^-1^)	y = 4.6346x + 102.69	0.987	0.975 (0.971)	0.00000012
TAG yield ^b^ (mg·L^-1^)	y = 5.3286x + 6.0908	0.987	0.974 (0.971)	0.00000012
Biodiesel yield ^b^ (mg·L^-1^)	y = 3.2342x + 38.003	0.997	0.993 (0.993)	0.00000000

Note: The superscripts a and b, respectively, indicate the fluorescence intensity (FI, a.u.) used in the regression analysis from the values at cell densities of 1.0×10^7^ and 6.0–8.5×10^7^ cells·mL^-1^ in the stained *N*. *oculata* suspension. *r*, correlation coefficient; *R*^*2*^, determination coefficient; TL, total lipids; TAG, triacylglycerols; Biodiesel yield, total FAMEs yield. The letter x and y in the regression equations, respectively, indicate the value of FI and the relevant regression variables. The values in the brackets of *R*^*2*^ column denote the adjusted *R*^*2*^.

### Cellular lipid bodies and ultra-microstructure

**Fig 6:**
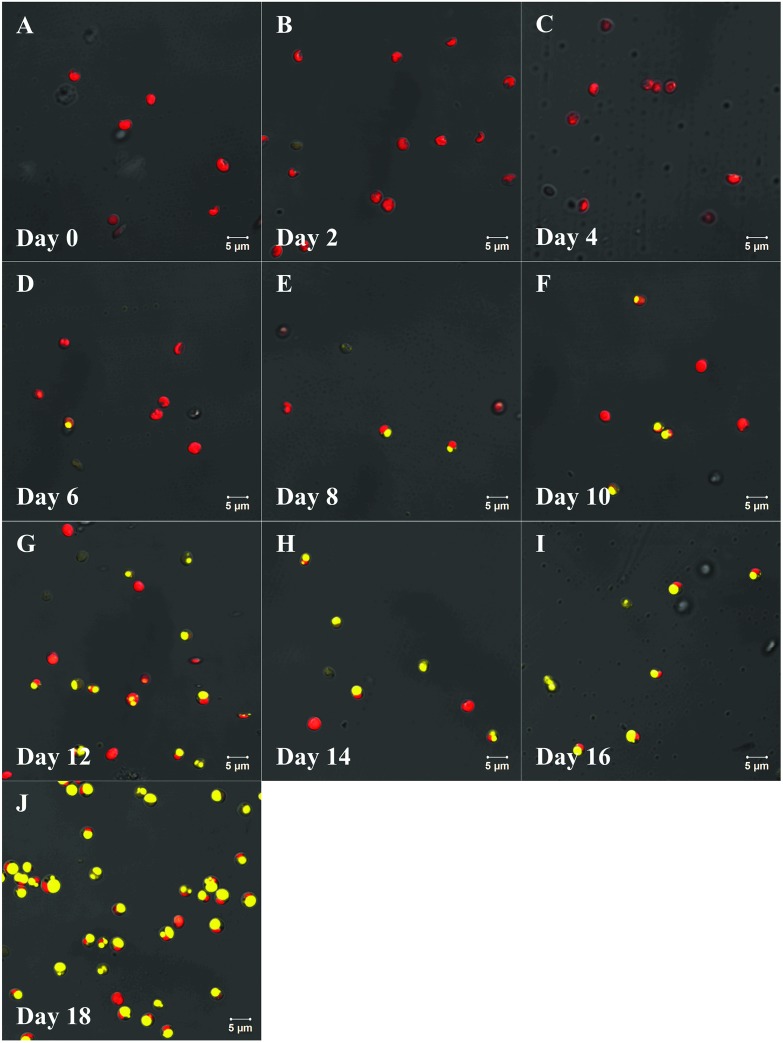
The intensity variation of intracellular lipid bodies stained with Nile red confirmed the lipid accumulation in *N*. *oculata* during long-term MFCS. This presents the confocal laser scanning micrographs (1260×) of *N*. *oculata* during multi-factor collaborative stresses (MFCS; i.e., 360 μmol·m^-2^·s^-1^ of high irradiation, nitrogen deficiency and 6.72 mg Fe·L^-1^ of iron supplementation). (a)–(j), the micrographs on day 0 to 18. The intracellular chloroplast and lipid body show red auto-fluorescence and yellow fluorescence, respectively. Scale bars = 5 μm.

*N*. *oculata* accumulated lipids in the form of lipid bodies (LBs), also called lipid droplet. The LBs of *N*. *oculata* stained with NR gave a strong yellow fluorescence by LSCM, while the chloroplasts showed a red auto-fluorescence (Fig 6). As a result, in situ specific fluorescent staining of living cells made it easy to locate the LBs of *N*. *oculata*. The fluorescence of the LBs was first captured on day 6 (Fig 6D), with 33.70% DW of TL content. Then, the accumulation rate of cellular lipids was augmented. Meanwhile, the number and size of the LBs in a single cell gradually increased, as well as its FI. However, only a single large yellow fluorescent spot could be observed in most *N*. *oculata* cells (Fig 6J). Nevertheless, the FI and the size of chloroplast decreased step by step. Thus, the cellular lipids of *N*. *oculata* gradually increased during MFCS, which was consistent with the results obtained by the gravimetric method.

**Fig 7:**
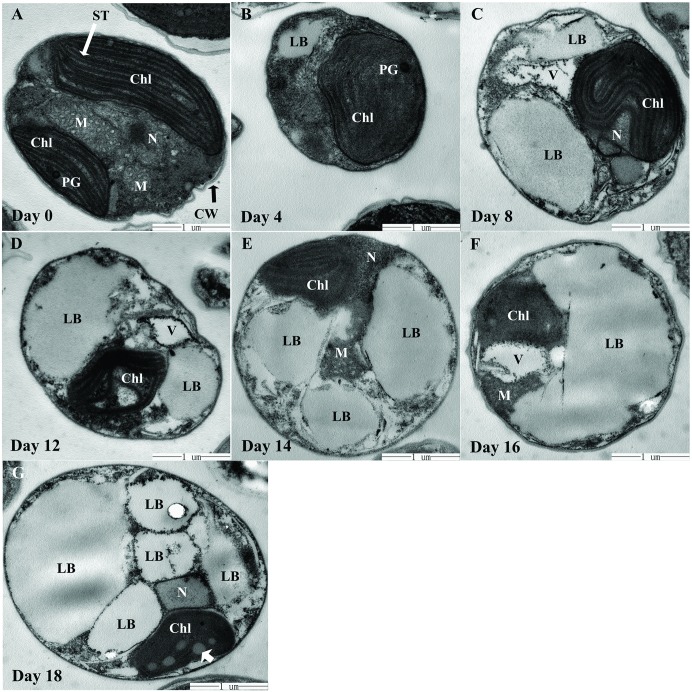
Cellular ultrastructure variation of *N*. *oculata* during long-term MFCS. This presents the transmission electron micrographs (23000×) of *N*. *oculata* during multi-factor collaborative stresses (MFCS; i.e., 360 μmol·m^-2^·s^-1^ of high irradiation, nitrogen deficiency and 6.72 mg Fe·L^-1^ of iron supplementation). (a), the cell on day 0 with few lipid bodies and normal organelle shape, including distinct stacks of thylakoids in chloroplast. (b), minute lipid bodies begin to emerge. (c), lipid bodies enlarge and large vacuoles are developed to degrade macromolecules. (d), (e), the obvious atrophy and degradation of chloroplast and other organelles; the accumulation of lipid bodies results in an increasingly full and round cell. (f), the fusion of some lipid bodies into a larger one. (g), several large lipid bodies oppress the protoplast content onto the cell wall on day 18; the poor chloroplast structure with impaired thylakoid membranes and small primary lipid bodies in the chloroplast (arrowhead). LB, lipid body; Chl, chloroplast; N, nucleus; M, mitochondrion; V, vacuole; PG, plastoglobulus; ST, stacks of thylakoids; CW, cell wall. Scale bars = 1 μm.

As shown by TEM, *N*. *oculata* cells had a spherical or oval shape, a diameter of approximately 2–4μm, a thin cell wall, an oval or cupulate chloroplast, several mitochondria, and a nucleus (Fig 7). In addition, pyrenoids or starch granules were not found. The average cellular volume increased somewhat after stress cultivation, but no thickening of the cell wall was observed.

With stress, the size and number of LBs in most cells increased progressively, filling the majority of the cellular space. Some of them merged into one large LB in a cell, resulting in an increasingly full cell. The cross-sectional area of the LBs made up approximately 60–70% of that of one single cell on day 18, and the diameter of the LB was 0.5–2μm (Fig 7G). This reflected the TL accumulation process of *N*. *oculata* during the course of MFCS. The long striped thylakoids were clearly visible and ran through the chloroplast at the beginning of the experiment (Fig 7A). As the stress progressed, the volume of the chloroplast apparently decreased (Fig 7D), and the thylakoid structure was increasingly fuzzy and eventually disintegrated, as well as other organelles (Fig 7G). Moreover, vacuoles began to emerge from day 4.

## Discussion

### Pigment alteration

*Nannochloropsis* is a promising source of commercially valuable pigments [24]. Its pigment composition is mostly Chl *a* with a small amount of carotenoids under favourable conditions. The adjustment of pigment composition is a mechanism for microalgae to adapt to environmental stresses [9, 25, 26]. Both high irradiation and nitrogen deficiency can cause cell yellowing (Fig 2F) by reducing chlorophyll content and increasing carotenoids proportion simultaneously (Fig 2A, 2C and 2D). One reason for that is the large amount of reactive oxygen species (ROS) generated under environmental stresses [27]. This is because pigments, especially chlorophyll, are very sensitive to oxidation reactions. However, carotenoids can protect against oxidative stress and dissipate excess excitation energy [28]. Another reason is that chlorophyll, which is rich in nitrogen, can be degraded heavily under nitrogen starvation (Figs 2 and 7), thus providing a nitrogen source for cell metabolism and biomass accumulation. Therefore, *N*. *oculata* started to enter the plateau phase until day 4 and the decline phase was yet to come on day 18 (Fig 1). Jiang *et al*. [29] also found similar results. However, confronted with the prolonged lack of nitrogen resources, the synthesis of nitrogen compounds, such as nucleic acid and protein, will be restricted and thus affect biomass production.

### The content changes of total lipids, protein and total sugar

MFCS could regulate *N*. *oculata* metabolism over a short duration (i.e., 4–12 d): more carbon was distributed into carbohydrates and lipids, protein synthesis was blocked and growth was limited (Figs 1 and 3). Carbohydrate metabolism was active—total sugar content increased at first, but then fluctuated within a small range (Fig 3C). While lipids have a higher energy density than carbohydrates, carbohydrates would be further converted into lipids by central carbon metabolism (e.g., TCA cycle) when carbohydrate reserves reach a certain threshold. It thus indicates that there are differential storage purposes between carbohydrates and lipids in some microalgae, carbohydrates as the preferentially synthesized and mobilized reserve and lipids representing long term storage in case of prolonged stress [2, 30]. In addition, the substantial degradation of cellular protein in *N*. *oculata* under MFCS (Fig 3B) may benefit the remobilization and redistribution of cellular nitrogen, such as the transformation from non-essential proteins to essential proteins, similar to the findings reported by Nicola Louise *et al*. [31] and Li *et al*. [32].

When cultured in favourable conditions (e.g., standard f/2 medium), *N*. *oculata* has a lipid content of about 25% (DW) [33]. This study and our high irradiation experiment (unpublished) found that both high light and nitrogen deficiency (more likely) collaboratively enhanced the lipid accumulation in *N*. *oculata*. High light can remodel the metabolite allocation in *Nannochloropsis* cells, especially for primary carbon partitioning between cell organelles. Such a regulation triggers a strong accumulation of TAGs in endoplasmic reticulum at the expensive of chloroplast, together with the up-regulation of genes involved in lipid biosynthesis [34, 35]. It is generally believed that under high light stress, excess electrons, produced by the electron transport chain in photosynthesis, will trigger the overproduction of ROS, thus limiting photosynthesis [27]. Yet, *de novo* FA synthesis can consume excess electrons, thereby alleviating photo-inhibition and photo-damage [28]. Thus, the amassing of TAGs is a mechanism used by some microalgae to adapt to high light stress [25, 36–38]. Many studies show that a high concentration of iron ions can promote not only the efficiencies of chlorophyll synthesis and photosynthesis but also lipid accumulation [14, 33, 39]. According to the nitrogen-iron-temperature combined culture experiment, *N*. *oculata* under 6.72 mg Fe·L^-1^ had a high TL content (53%) and the highest lipid production (142 mg·L^-1^) [14]. There are direct action sites for iron in the electron transport chains in chloroplasts and mitochondria, which affect photosynthesis and respiration, thus affecting carbon storage and lipid reserves [33, 39].

### Lipid class composition, FA profile, and biodiesel properties

High NL content (mainly TAGs) is an important advantage for microalgae as biodiesel feedstock [8]. There is a low content ratio of NL/PL for *N*. *oculata* under non-stressful conditions [14, 33]. As the stresses progressed, the NL proportion of *N*. *oculata* increased dramatically (Fig 4A and S1 Fig). Microalgae can rapidly remobilize TAG reserves stored in LBs for cell proliferation in a more favourable environment; for example, nitrogen replenishment triggers LB degradation [26, 40]. In addition, there were 8.83 times as many TAGs proportionally on day 18 as in the beginning, while the other lipid classes decreased correspondingly (Fig 4A). Perhaps there are ways for redundant PLs or other lipid classes to transform into TAGs [7].

Nitrogen deficiency can result in the declining generation of chloroplasts, which leads to lower demands for membrane structural components (e.g., PLs and glycerolipids) rich in PUFA [14, 26, 30, 41]. In this study, the PUFA proportion gradually declined as the stress progressed, while the proportions of SFA and MUFA increased accordingly, their sum increasing from 63.56% to 92.18% (Table 1). The FA profile of *N*. *oculata* cells here is the opposite of that under non-stressful conditions [14, 33]. Excessive high light can result in the oxidative damage of PUFA, but promote the synthesis of SFA beneficial to consume excess photo-energy to alleviate photo-damage [28, 38]. Both C16:0 and C18:1, for example, are important precursors of TAG synthesis [42], and their proportions and absolute contents all remarkably increased (Table 1 and S1 Fig).

Microalgae biodiesel exhibits excellent properties in environmental protection, lubrication, low-temperature start-up and renewability [1]. However, little is known about the effect of environmental stresses on the properties of microalgae biodiesel. In view of the effect of FA profile on CFPP and IV, both SFA and PUFA in biodiesel should be as low as possible [43]. Biodiesel feedstock with high quality should have a high MUFA content, especially straight-chain C16 or C18 MUFA [44]. In this study, as the stress progressed, the proportions of SFA and MUFA increased, while the PUFA proportion decreased (Table 1). These changes, therefore, resulted in the significant improvement of IV and CN (Table 2). However, the augmentation of SFA proportion led to somewhat poor CFPP, which still met the biodiesel standards in relevant countries (S2 Table). This study indicated that environmental stresses could benefit the properties of *N*. *oculata* biodiesel.

### The use of fluorescent Nile red for neutral lipid measurement

Previous reports often qualitatively describe lipids by the NR staining method [45–47]. By combining the conventional methods and the fluorometric staining using NR, this study identified linear regression equations with high goodness of fit between FI and the oil-yielding performance indexes (i.e., TL content, TAG content, and biodiesel production) (Table 3). As a result, we could apply these equations in the quantification determination of oil-yielding performances of *N*. *oculata* at different growth phases during MFCS, without need of pre-concentration.

The results obtained in this novel way were comparable to those determined by traditional methods (Table 3). This approach is easy, real-time and economic for microalgae NL quantification compared with conventional gravimetric methods which require approximately 0.2g dry biomass of microalgae to determine lipid content [16]. Furthermore, multifunctional fluorescent ELISA can be used in this method for the large-scale screening of oleaginous microalgal strains and the optimization of culture conditions.

### Cellular ultramicrostructural changes

The LBs were 0.5–2μm in diameter (Fig 7), similar to the reports for plant seeds and algae [29]. The LBs of *N*. *oculata* were relatively independent, stable and integrated in shape. As the stresses progressed, the LBs gradually filled most of the cell space and the cell became plump, which is similar to the findings of Jiang *et al*. [29]. Surface proteins of LBs (e.g., oleosin), abundant in microalgae and plant seeds, could help maintain the stability and integrity of LB shape [40] and may participate in cell signal transmission, stress response and lipid metabolism [48]. The ontogeny of LBs is not very clear so far. It is thought that LBs, as TAG deposit, are firstly generated by two lobes of the endoplasmic reticulum, and then develop into a discontinuous organelle, attached or not to the endoplasmic reticulum membrane [49].

Autophagy is a vital pathway for plants to recycle cytoplasmic contents in the vacuoles upon environmental stress [26, 50]. *N*. *oculata* generated large vacuoles containing membranous inclusions and isolated organelles (Fig 7C and 7F). Therefore, the cellular destruction of *N*. *oculata* could be related to the autophagy induced by MFCS. We found that thylakoid membrane degradation occurred in *N*. *oculata* (Fig 7), in agreement with the results from the chemical extraction method (Fig 2, reduced chlorophyll level). Both Yang *et al*. [51] and Iwai *et al*. [42] reported the similar findings. This result may imply the progressive decline of the chlorophyll antenna size to adapt to environmental stresses.

Our study provides evidence that MFCS could significantly promote the oil-yielding performance of *N*. *oculata* in a short duration (i.e., 4–12 d). This will help reveal its adaptation mechanisms to environmental stresses, and better understand the manipulation of metabolic pathway to enhance the production of algal oil in oleaginous microalgae.

## Supporting information

S1 Fig Variations in the neutral and polar lipid compositions of *N*. *oculata* during long-term MFCS. MFCS means multi-factor collaborative stresses (i.e., 360 μmol·m^-2^·s^-1^ of high irradiation, nitrogen deficiency and 6.72 mg Fe·L^-1^ of iron supplementation). NL, neutral lipid; PL, polar lipid. Different small letters for neutral lipid column denote significant differences among different stress durations at *P*<0.05. (means±SD of three replicates).
(TIF)

S2 Fig Variations in the relative content and relative production of cellular Neutral Lipids (NLs) in *N*. *oculata* during long-term MFCS. MFCS means multi-factor collaborative stresses (i.e., 360 μmol·m^-2^·s^-1^ of high irradiation, nitrogen deficiency and 6.72 mg Fe·L^-1^ of iron supplementation). The relative quantification of NL was represented by the fluorescence intensity (FI, a.u.) of cellular NL stained with Nile red in each day, as the stained *N*. *oculata* suspension was diluted to a cell density of 1.0×10^7^ cells·mL^-1^ (for the relative content of NL) or being at its original cell density of 6.0–8.5×10^7^ cells·mL^-1^ (for the relative production of NL). Different small letters for the relative content of NL mean significant differences among different stress durations at *P*<0.05. (means±SD of three replicates).
(TIF)

S1 Table Variation in the fatty acid profile of *N*. *oculata* during long-term MFCS. (DOCX)

S2 Table Quality items of biodiesel standards in different countries. (DOCX)
