# Supplementary figures and images for: Long-duration effect of multi-factor stresses on the cellular biochemistry, oil-yielding performance and morphology of Nannochloropsis oculata
Source: PLoS One. 2017 Mar 27;12(3):e0174646. doi: 10.1371/journal.pone.0174646 (PMC5367823; doi:10.1371/journal.pone.0174646)

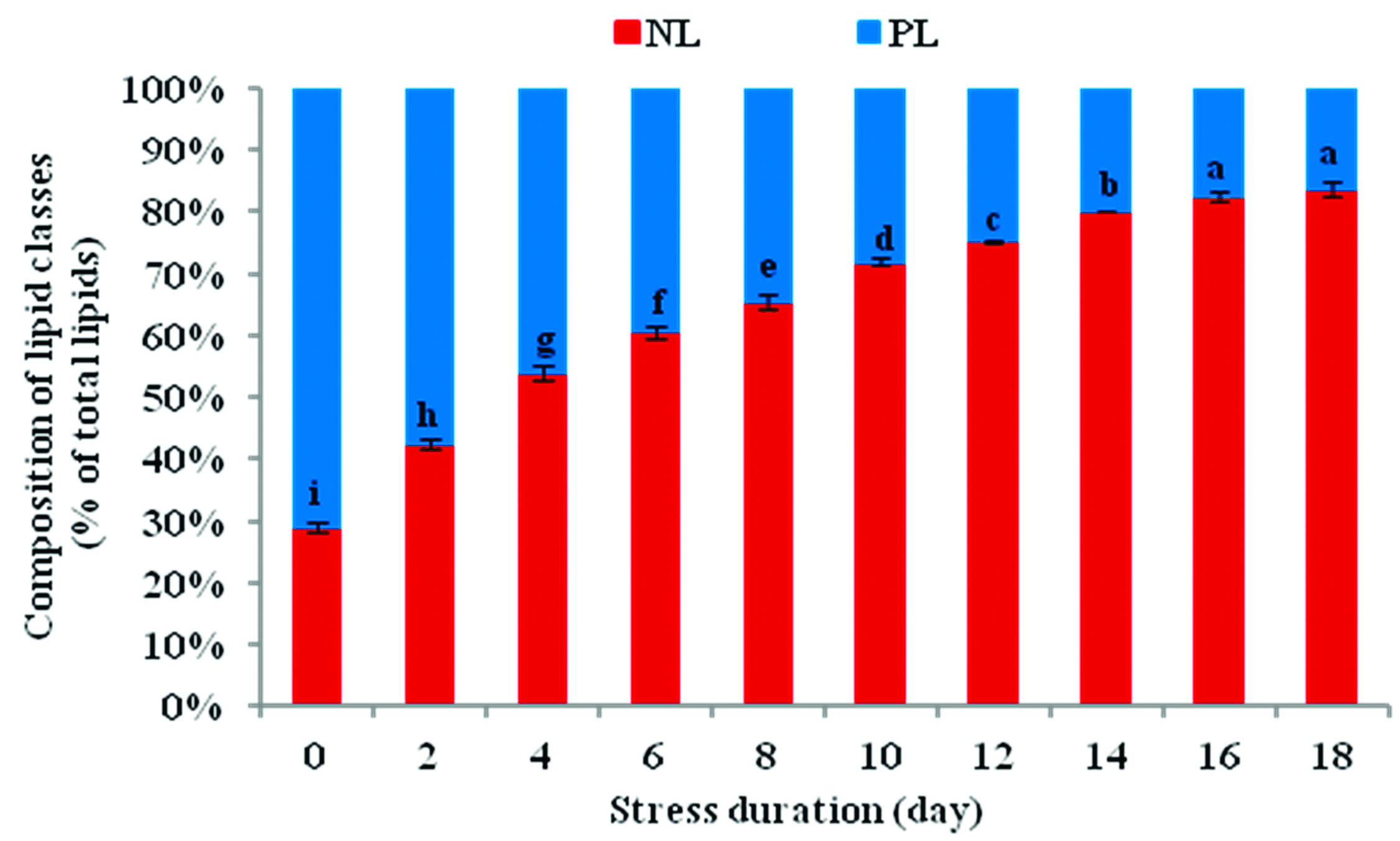

Supplement: S1 Fig — MFCS means multi-factor collaborative stresses (i.e., 360 μmol·m-2·s-1 of high irradiation, nitrogen deficiency and 6.72 mg Fe·L-1 of iron supplementation). NL, neutral lipid; PL, polar lipid. Different small letters for neutral lipid column denote significant differences among different stress durations at P<0.05. (means±SD of three replicates). (TIF) [file pone.0174646.s001.tif]

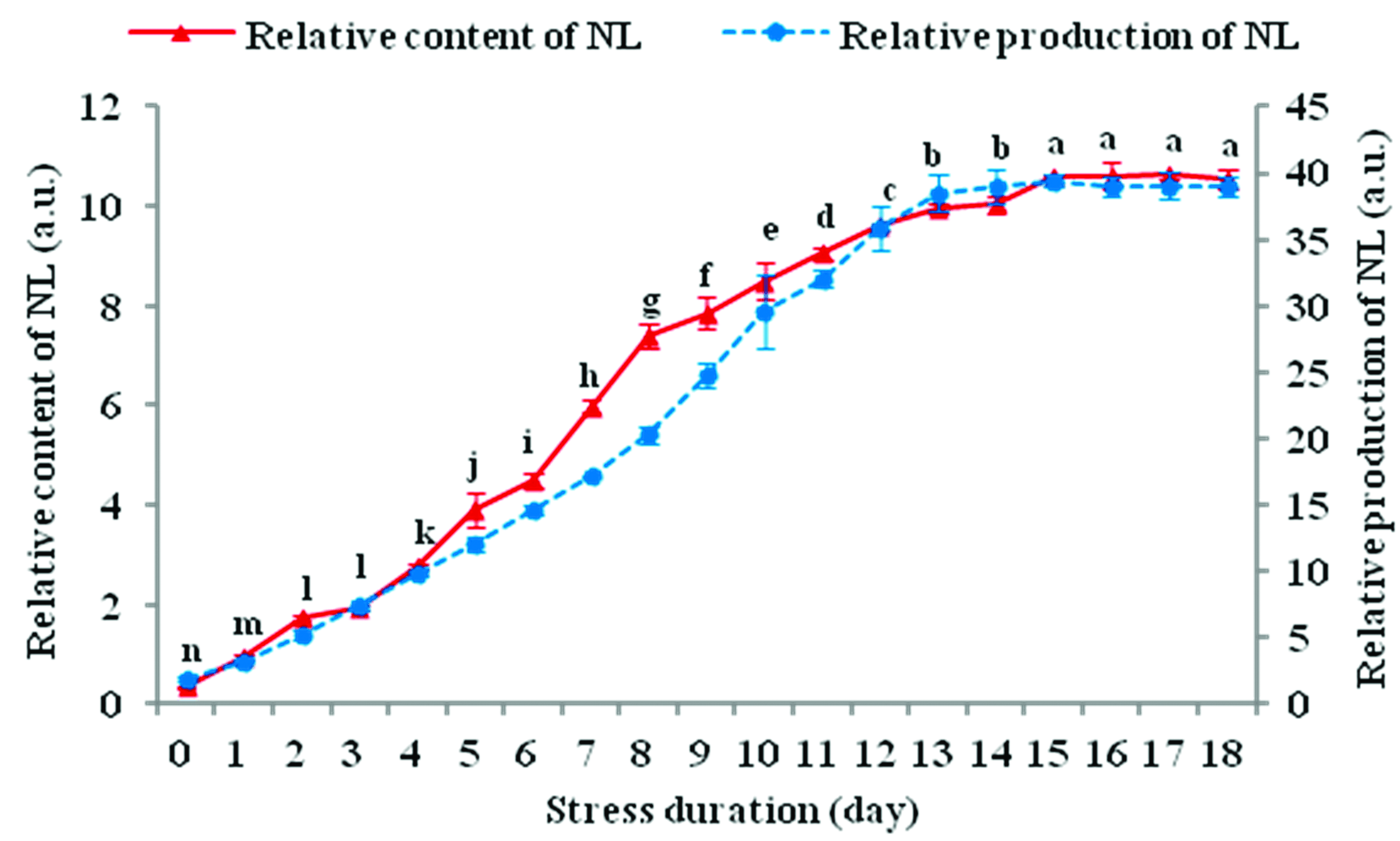

Supplement: S2 Fig — MFCS means multi-factor collaborative stresses (i.e., 360 μmol·m-2·s-1 of high irradiation, nitrogen deficiency and 6.72 mg Fe·L-1 of iron supplementation). The relative quantification of NL was represented by the fluorescence intensity (FI, a.u.) of cellular NL stained with Nile red in each day, as the stained N. oculata suspension was diluted to a cell density of 1.0×10^7^ cells·mL-1 (for the relative content of NL) or being at its original cell density of 6.0–8.5×10^7^ cells·mL-1 (for the relative production of NL). Different small letters for the relative content of NL mean significant differences among different stress durations at P<0.05. (means±SD of three replicates). (TIF) [file pone.0174646.s002.tif]
