# Supplementary material for: Long-duration effect of multi-factor stresses on the cellular biochemistry, oil-yielding performance and morphology of Nannochloropsis oculata
Source: PLoS One. 2017 Mar 27;12(3):e0174646. doi: 10.1371/journal.pone.0174646 (PMC5367823; doi:10.1371/journal.pone.0174646)
Supplement: S1 Table — (DOCX) [file pone.0174646.s003.docx]

**S1 Table. Variation in the fatty acid profile of *N. oculata* during long-term MFCS.**

| Fatty acids(mg·g^-1^ DW) | Stress duration (day) | | | | | | | | | |  |
| --- | --- | --- | --- | --- | --- | --- | --- | --- | --- | --- | --- |
|  | 0 | 2 | 4 | 6 | 8 | 10 | 12 | 14 | 16 | 18 | |
| 14:0 | 2.99±0.23 | 2.87±0.24 | 3.37±0.34 | 4.74±0.40 | 5.07±0.22 | 5.30±0.07 | 5.84±0.14 | 6.11±0.70 | 6.26±0.15 | 5.84±0.54 | |
| 16:0 | 24.99±0.43 | 38.90±3.09 | 48.70±3.42 | 67.76±1.74 | 82.26±2.35 | 93.30±2.44 | 107.17±1.51 | 107.53±10.97 | 109.50±1.40 | 105.10±0.68 | |
| 16:1n7 | 27.48±1.41 | 34.37±1.18 | 38.17±7.97 | 45.05±1.00 | 55.70±1.79 | 66.64±1.64 | 74.34±1.92 | 72.94±7.85 | 75.95±0.39 | 75.27±2.64 | |
| 18:0 | 2.38±0.01 | 1.82±0.13 | 2.03±0.14 | 2.67±0.20 | 3.04±0.21 | 3.44±0.25 | 4.15±0.26 | 4.20±0.61 | 4.65±0.20 | 3.69±0.33 | |
| 18:1n9 | 6.58±0.05 | 11.04±1.48 | 12.39±1.12 | 19.62±0.35 | 26.92±0.75 | 35.47±2.00 | 40.78±1.54 | 44.09±4.06 | 49.22±0.70 | 46.17±0.30 | |
| 18:2n6 | 6.18±0.04 | 5.03±0.26 | 6.15±2.40 | 5.00±0.04 | 4.91±0.12 | 4.94±0.28 | 4.60±0.33 | 4.46±0.25 | 4.74±0.16 | 4.06±0.35 | |
| ARA | 11.85±0.68 | 10.17±0.47 | 9.10±0.00 | 9.94±0.53 | 9.22±0.55 | 8.27±0.43 | 8.92±0.49 | 8.95±0.70 | 8.73±0.26 | 6.76±0.72 | |
| EPA | 25.88±0.25 | 18.75±0.94 | 16.76±1.60 | 17.31±0.59 | 15.77±0.97 | 15.12±0.89 | 15.67±0.68 | 15.30±1.39 | 15.68±0.54 | 12.32±1.95 | |
| Others | 1.87±0.20 | 2.24±0.18 | 2.29±0.01 | 2.74±0.10 | 2.90±0.16 | 3.06±0.06 | 3.04±0.04 | 3.04±0.23 | 3.17±0.31 | 2.73±0.08 | |
| ∑16C | 52.47±0.98 | 73.27±1.90 | 86.87±11.39 | 112.81±2.68 | 137.96±4.02 | 159.95±4.05 | 181.51±0.41 | 180.47±18.70 | 185.45±1.01 | 180.37±1.96 | |
| ∑18C | 15.14±0.02 | 17.89±1.35 | 20.56±3.66 | 27.29±0.56 | 34.88±0.77 | 43.85±2.49 | 49.54±1.46 | 52.75±4.59 | 58.61±1.06 | 53.92±0.38 | |
| ∑20C | 37.74±0.42 | 28.93±1.41 | 25.86±1.61 | 27.25±1.11 | 25.00±1.35 | 23.39±1.09 | 24.58±1.17 | 24.25±2.06 | 24.42±0.29 | 19.08±2.68 | |
| ∑SFA | 31.46±0.50 | 45.07±3.62 | 55.73±3.11 | 77.29±1.76 | 92.62±2.64 | 104.44±2.68 | 119.52±1.40 | 120.23±12.21 | 122.93±1.64 | 116.78±0.55 | |
| ∑MUFA | 34.84±1.39 | 46.17±0.31 | 51.20±9.21 | 65.29±0.86 | 83.27±2.46 | 102.77±3.55 | 115.81±3.44 | 117.69±11.97 | 125.82±0.42 | 122.02±2.93 | |
| ∑PUFA | 43.91±0.38 | 33.96±1.15 | 32.01±4.01 | 32.25±1.10 | 29.91±1.42 | 28.33±1.30 | 29.19±1.50 | 28.71±2.09 | 29.15±0.13 | 23.14±3.03 | |

Note: MFCS means multi-factor collaborative stresses (i.e., 360 μmol·m^-2^·s^-1^ of high irradiation, nitrogen deficiency and 6.72 mg Fe·L^-1^ of iron supplementation). Other fatty acids (i.e., Others in this table) are C12:0, C15:0, C17:0 and C17:1n7, all of whose contents are less than 1% of total fatty acids. ARA, arachidonic acid (C20:4n6); EPA, eicosapentaenoic acid (C20:5n3); SFA, saturated fatty acids; MUFA, monounsaturated fatty acids; PUFA, polyunsaturated fatty acids. (means±SD of three replicates).
