# Supplementary material for: Long-duration effect of multi-factor stresses on the cellular biochemistry, oil-yielding performance and morphology of Nannochloropsis oculata
Source: PLoS One. 2017 Mar 27;12(3):e0174646. doi: 10.1371/journal.pone.0174646 (PMC5367823; doi:10.1371/journal.pone.0174646)
Supplement: S2 Table — (DOCX) [file pone.0174646.s004.docx]

**S2 Table. Quality items of biodiesel standards in different countries.**

| Quality items | USA | European Union | Germany | China |
| --- | --- | --- | --- | --- |
|  | ASTM  D6751-2010*^a^* | UNE-EN 14214-2008*^b^* | DIN  51606-1997*^c^* | GB/T  20828-2007*^d^* |
| Vis (mm^2^·s^-1^) | 1.9～6.0 | 3.5～5.0 | 3.5～5.0 | 1.9～6.0 |
| IV (gI_2_·100g^-1^) | - | ≤120 | ≤115 | - |
| CN | 47-65 | ≥51 | ≥49 | ≥49 |
| CFPP (℃) | 0/-10/-20 | 0/-15 | 0/-10/-20 | Report |

Note: Vis, viscosity; IV, iodine value; CN, cetane number; CFPP, cold filter plugging point.

*^a^*ASTM D6751 (2010). Standard specification for biodiesel fuel lend stock (B100) for middle distillate fuels.

*^b^*UNE-EN 14214 (2008). Automotive fuels-fatty acid methyl esters (FAME) for Diesel Engines-Requirement Methods.

*^c^*Deutsche Norm DIN E 51606 (1997). Dieselkraftstoff aus Fettsäuremethylester (FAME).

*^d^*GB/T 20828 (2007). Biodiesel blend stock (BD100) for diesel engine fuels.
